# Retraction: Unleashing the behavioral factors affecting the decision making of Chinese investors in stock markets

**DOI:** 10.1371/journal.pone.0311395

**Published:** 2024-11-11

**Authors:** The PLOS ONE Editors

The *PLOS ONE* Editors retract this article [1] because it was identified as one of a series of submissions for which we have concerns about authorship, ethics approval, integrity of the underlying data, the reliability of the published results, and peer review. We regret that the issues were not identified prior to the article’s publication.

GRM did not agree with the retraction. YX either did not respond directly or could not be reached.
